# 105 Burn Injury Severity: Proposed Definitions Based on the National Burn Research Dataset

**DOI:** 10.1093/jbcr/irae036.104

**Published:** 2024-04-17

**Authors:** Jason Heard, Yunyi Ren, Soman Sen, Kathleen S Romanowski, Tina L Palmieri, David G Greenhalgh

**Affiliations:** University of California - Davis Hosp, Sacramento, CA; UC Davis Health, Sacramento, California; UC Davis, Sacramento, CA; UC Davis and Shriners Children's Northern California, Sacramento, CA; Shriners Hospital Northern California, Sacramento, CA; University of California - Davis Hosp, Sacramento, CA; UC Davis Health, Sacramento, California; UC Davis, Sacramento, CA; UC Davis and Shriners Children's Northern California, Sacramento, CA; Shriners Hospital Northern California, Sacramento, CA; University of California - Davis Hosp, Sacramento, CA; UC Davis Health, Sacramento, California; UC Davis, Sacramento, CA; UC Davis and Shriners Children's Northern California, Sacramento, CA; Shriners Hospital Northern California, Sacramento, CA; University of California - Davis Hosp, Sacramento, CA; UC Davis Health, Sacramento, California; UC Davis, Sacramento, CA; UC Davis and Shriners Children's Northern California, Sacramento, CA; Shriners Hospital Northern California, Sacramento, CA; University of California - Davis Hosp, Sacramento, CA; UC Davis Health, Sacramento, California; UC Davis, Sacramento, CA; UC Davis and Shriners Children's Northern California, Sacramento, CA; Shriners Hospital Northern California, Sacramento, CA; University of California - Davis Hosp, Sacramento, CA; UC Davis Health, Sacramento, California; UC Davis, Sacramento, CA; UC Davis and Shriners Children's Northern California, Sacramento, CA; Shriners Hospital Northern California, Sacramento, CA

## Abstract

**Introduction:**

Overall care of the burn injured patient has improved substantially leading to improved survival and outcomes. However, despite these improvements, how burn severity is classified based on total body surface area burned (TBSA) and depth has not changed. No recent work has employed data-driven methods to distinguish burns as minor, moderate, severe, or massive. Our aim was to leverage the National Burn Repository to develop data driven definitions of burn severity.

**Methods:**

We used the American Burn Association (ABA) Burn Research Dataset encompassing all burn admissions from contributing burn centers from 2008-2018. Exclusion criteria were aged < 18 years, death, or hospice care within 7 days of admission, or burn etiology of cold injury, trauma, electrical burns, or no cutaneous injury. Latent class analysis (LCA) was performed using the following categorized patient variables: length of stay, ICU length of stay, number of complications, bedside procedures (central line, arterial line, feeding tube placement, and bronchoscopy), amputation, tracheostomy, excisions, grafting, and transfusions. Hierarchical clustering was then applied to the most severe burn group identified through LCA to further stratify severe from massive burn injuries.

**Results:**

There were 112,297 unique records included in analysis. The LCA identified three clusters termed minor (N=63,637), moderate (N=37,320) and severe (N=11,340). The severe group was further split into two groups: severe (N=9,975) and massive (N=1365) using HCA. The mean (SD) total TBSA burned in the groups were 4.2(4.9) in mild, 7.8(7.9) in moderate, 23.4(18) in severe and 39.8(22.4) in massive group. The mean rBaux score in the mild group was 48.2(18.2), 55.0(19.5) in moderate, 80.3(22.8) in severe and 94.5(24.7) in the massive group.

**Conclusions:**

While burn injuries exist on a continuum of severity there are advantages to grouping burn injury severity based on size. Objective and modern categorization can not only help triage patients with burn injuries, but also will be helpful in burn research. There are variable definitions for severe and massive burn injuries in the literature which leads to ambiguity when comparing studies on this unique patient population. This analysis supports a definition of < 10% TBSA for minor burns, 10-20% TBSA for moderate burns, 20-40% TBSA for severe burns and >40% TBSA for massive burns.

**Applicability of Research to Practice:**

This research can help burn and non-burn centers triage patients based on expected resource utilization. However, this research will be most applicable to research that aims to study patients based on burn severity now that more homogenous groups have been defined.

